# Preliminary study on the preparation of antler powder/chitosan/β-glycerophosphate sodium/polyvinyl alcohol porous hydrogel scaffolds and their osteogenic effects

**DOI:** 10.3389/fbioe.2024.1421718

**Published:** 2024-06-26

**Authors:** Kudelaiti Abudukelimu, Aikepaer Aierken, Ailifeire Tuerxuntayi, Yilizhati Yilihamu, Saierdaer Abulizi, Duolikun Wufuer, Hongbin Dong

**Affiliations:** ^1^ Department of Prosthodontics, The First Affiliated Hospital (Affiliated Stomatological Hospital) of Xinjiang Medical University, Urumqi, China; ^2^ People’s Hospital of Xinjiang Uygur Autonomous Region, Urumqi, China; ^3^ College of Engineering Science, University of Chinese Academy of Sciences, Beijing, China

**Keywords:** antler powder, chitosan, sodium β-glycerophosphate, polyvinyl alcohol, bone regeneration, bone tissue scaffold

## Abstract

**Introduction:** The production of bone-like structural scaffolds through bone tissue engineering technology is a promising method for bone regeneration to repair bone defects. Deer antler, an easily harvested and abundantly sourced initial bone tissue structure, resembles the composition and structure of human cancellous bone and can serve as a new material for allogeneic bone transplantation.

**Methods:** This study involved the preparation and characterization of antler powder/chitosan/β-glycerophosphate sodium/polyvinyl alcohol (AP/CS/β-GP/PVA) porous hydrogel scaffolds to verify their material properties and osteogenic mechanisms. The microstructure, hydrophilicity, and mechanical properties of the scaffolds were studied using Scanning Electron Microscopy (SEM), contact angle measurement, and a universal material testing machine. The interactions between the various components were investigated using Fourier-Transform Infrared Spectroscopy (FTIR). Biocompatibility, osteogenic properties, and expression of osteogenesis-related proteins of the scaffolds were evaluated through Cell Counting Kit-8 (CCK-8) assays, alkaline phosphatase staining, Alizarin Red staining, live/dead cell staining, and Western blot analysis.

**Results:** The results showed that as the content of deer antler powder increased, both the hydrophilicity and mechanical properties of the scaffold materials improved, while the porosity slightly decreased with an increase in deer antler powder content. Cell culture experiments demonstrated that scaffolds with a higher proportion of deer antler powder were beneficial for the proliferation and differentiation of mouse pre-osteoblast (MC3T3-E1) cells, with the scaffolds containing 10% and 8% deer antler powder showing the best effects. The upregulation of RUNX2, OCN, OSX, and OPN protein expression may promote differentiation.

**Discussion:** Therefore, the AP/CS/β-GP/PVA hydrogel scaffolds have the potential to become a promising biomaterial for bone tissue engineering.

## Graphical Abstract

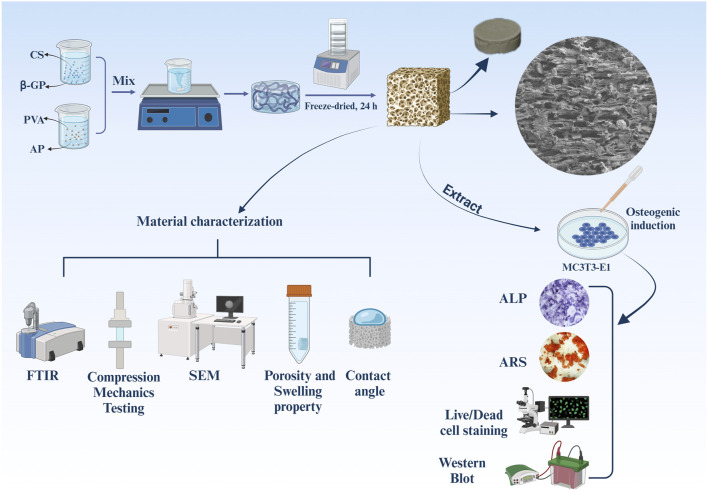

## 1 Introduction

The amount of available alveolar bone within the mouth is key to the selection of treatment methods and treatment approaches in oral rehabilitation. Clinically, the loss of alveolar bone volume in patients can be caused by reasons such as tooth extraction, severe periodontitis, post-oral tumor surgery, cleft lip and palate, and congenital developmental abnormalities (Bouziane et al., 2020; Luo et al., 2023; Zheng et al., 2023; Huang et al., 2024). The absence of adequate horizontal and vertical bone is a problem that can affect the survival rate of dental restoration (Santos et al., 2023; Sun et al., 2023). In response to such issues, clinicians resort to bone grafting to enhance the bone level (Zhao et al., 2021). Autologous bone grafting, with its superior osteogenic, osteoinductive, and osteoconductive properties compared to allogeneic bone grafts, has become the gold standard for preserving the level of alveolar bone and reconstructing bone defects (Gillman and Jayasuriya, 2021; Maevskaia et al., 2023). However, autologous bone grafting has drawbacks such as resorption of the grafted area, insufficient bone volume in the donor area, vulnerability of the donor area to trauma, pain, infection, and sensory abnormalities (Bernardi et al., 2020). As for allogeneic bone grafting, it also faces potential challenges related to immune rejection, tissue compatibility, cross-transmission of diseases, as well as the biological or mechanical properties of the materials used (Kloss et al., 2023; Xu et al., 2021). Despite the advantages of both autologous and allogeneic bone grafting, concerns regarding their application have led to the development of many bone substitutes. An ideal bone substitute should provide biomechanical support and create a suitable microenvironment for cell adhesion, proliferation, and differentiation (Li, 2023).

Deer antlers are the only body part in mammals that can fully regenerate; once lost, they can completely grow back naturally (Chu et al., 2021). Antlers are renowned for their astonishing growth rate, increasing by 2–4 cm daily, making them one of the fastest-growing tissues in the animal kingdom (Li and Suttie, 2000). The remarkable growth rate of deer antlers is facilitated by the abundant blood supply provided by the highly vascularized velvet tissue on the antler surface and the pedicle at the antler base (Zhang et al., 2023a). During antlerogenesis, there is a rapid deposition of calcium and phosphate within a relatively short period. This process leads to the formation of a cancellous bone-like structure in the central portions of the antlers. Antlers share a similar composition with human cancellous bones and other long bones found in mammals (Steiner-Bogdaszewska et al., 2022). Deer antler powder is abundantly sourced and easy to harvest, providing a sustainable and cost-effective material for bone tissue engineering.

Polyelectrolyte complexes composed of chitosan and β-glycerophosphate can enhance the performance of individual polymers, increase the three-dimensional structure of scaffolds, improve cell adhesion, and enhance the biocompatibility of the material (Tong et al., 2022). Additionally, chitosan/β-glycerophosphate hydrogels at specific concentrations can promote the proliferation of bone marrow mesenchymal stem cells and have a good function in promoting tissue recovery (Ahmadi and de Bruijn, 2008). Currently, chitosan-β-glycerophosphate hydrogels, with their excellent material properties and similarity to the extracellular matrix, are widely used in tissue engineering (Badr et al., 2023; Arpornmaeklong et al., 2023; Zheng et al., 2022).

Polyvinyl alcohol (PVA) has attracted immense attention in biomedical applications owing to its inherent advantageous mechanical properties, low cytotoxicity and FDA approval for oral medicine usage (Cutiongco et al., 2016). Meanwhile, when polyvinyl alcohol (PVA) solutions are subjected to freeze-thaw cycles, microcrystals form as cross-linking nodes, promoting the cross-linking of hydrogels (Xu et al., 2020). Moreover, the hydroxyl groups (−OH) attached to the PVA main chain serve as sources of hydrogen bonds, improving the complexation process, thereby facilitating blending with other polymers (Lan et al., 2019).

This study aims to enhance the mechanical strength and biological activity of chitosan/β-glycerophosphate hydrogels by attempting to prepare a novel deer antler powder/chitosan/β-glycerophosphate/PVA hydrogel scaffold. It explores the chemical and physical properties, microstructure, mechanical properties, biocompatibility, and osteogenic activity of the deer antler powder/chitosan/β-glycerophosphate/PVA hydrogel scaffold.

## 2 Materials and methods

### 2.1 Materials

Antler powder (Tefeng Pharmaceutical Co., Ltd., Urumqi, China). PVA-1750 ± 50, Chitosan (degree of deacetylation, 85%), disodium β-glycerophosphate pentahydrate (Yuanye Bio-Technology Co., Ltd., Shanghai, China). Mouse pre-osteoblasts (MC3T3-E1 Subclone 14) cells (Service Biotechnology Co., Wuhan, China), Fetal bovine serum (FBS), alphaminimum essential medium (α-MEM), Phosphate buffered saline (PBS) and penicillin/streptomycin (Gibco, Invitrogen, Carlsbad, CA, United States). CCK8 kit and BCIP/NBT Alkaline Phosphatase Color Development Kit (Beyotime Biological Co., Ltd., Shanghai, China), Deionized water (UPR-IV-10 T, China). Deionized water was used in all experiments.

### 2.2 Fabrication of scaffolds

First, weigh 2.5 g of chitosan and add it to a 0.1 mol/L acetic acid solution. Stir the resulting solution with a magnetic stirrer until it is completely dissolved, which takes about 1 h. While stirring, gradually add 40 mL of 25% β-GP solution to obtain a uniform and transparent CS/β-GP liquid solution. Next, dissolve 8.0 g of PVA in 50 mL of deionized water and stir at 90°C for 4 h to prepare the PVA solution. Then disperse Antler powder in different contents (6.0, 8.0, and 10.0 g; 6%, 8%, and 10%) into the PVA solution, and stir for an additional 2 h. Mix these two solutions in a 1:1 ratio, continue stirring for 1 h, and then let it degas by resting. Pour the mixture into a sterile 24-well plate, with each well containing less than 1 mL of the solution. Seal the plate and freeze it at −80°C overnight, then freeze-dry for 24 h.

### 2.3 Fourier transform infrared spectroscopy

The interactions of Antler powder, chitosan, sodium β-glycerophosphate, and PVA in forming the polymer were studied using infrared spectroscopy, and compared with individual polymer samples. The Fourier Transform Infrared spectra were obtained using a Nicolet-6700 spectrometer (Thermo Scientific, America) in transmission mode from 4,000 to 450 cm^−1^, with a resolution of 4 cm^−1^ and 32 scans. Samples were mixed with KBr to prepare the FTIR samples.

### 2.4 Scanning electron microscope

Scanning Electron Microscopy (SEM, Jeol JSM-7610FPlus, Japan) was used to observe the microstructure of CS/PVA, 6%AP/CS/β-GP/PVA, 8%AP/CS/β-GP/PVA, and 10%AP/CS/β-GP/PVA hydrogel scaffolds. Before examination, each sample was sputtered with platinum (Pt).

### 2.5 Contact angle measurement

The hydrophilicity of the scaffolds was characterized by contact angle measurements using an interfacial rheometer (SL200KS, KINO, United States). A 5 μL droplet of water was placed on the surface of the sample film and photographed after 5 s. The angle of each droplet was measured and analyzed. Each test was conducted three times.

### 2.6 Swelling property

To evaluate the swelling properties of the scaffolds, a method for determining water absorption in phosphate buffered saline (PBS) was employed. The specific steps are as follows: First, the lyophilized hydrogel scaffolds (each with a diameter of 10 mm and thickness of 4 mm, with three samples per group) were initially weighed, recorded as W0. Then, these hydrogel scaffolds were fully immersed in 3 mL of PBS at pH 7.4 and placed in a constant temperature environment at 37°C to simulate *in vivo* conditions. At designated time points, the hydrogel scaffolds were removed from the PBS, surface moisture was gently wiped off, and they were immediately reweighed, recorded as W1. This process was continued until the weight of the hydrogel scaffolds stabilized, indicating that they had reached swelling equilibrium. The swelling rate S (%) is calculated using the following formula: Swelling rate (%) = W1-W0/W0 × 100%.

### 2.7 Porosity

To accurately calculate the porosity of scaffolds, this study employed the liquid displacement method. The specific steps of this method are as follows: First, immerse the hydrogel scaffolds (each with a diameter of 10 mm and a thickness of 4 mm, three samples per group) completely in anhydrous ethanol with a volume of V1. After 48 h, when the hydrogel scaffolds have fully absorbed the anhydrous ethanol and there are no bubbles on the surface of the liquid, record the volume as V2. Then, remove the scaffolds and measure the volume of the remaining anhydrous ethanol, V3. The porosity P (%) is calculated using the following formula: Porosity (%) = (V1–V3)/(V2–V3) × 100%.

### 2.8 Mechanical properties test

The maximum compressive strength of the scaffolds was tested using a CTM 8050 universal testing machine (Shie Qiang, China). The cylindrical scaffolds, with a diameter of 25 mm and a height of 10 mm, were compressed at a rate of 5 mm/min, using a pressure sensor with a maximum range of 2000 N.

### 2.9 Cell proliferation assay

The MC3T3-E1 cells were cultured in alpha minimum essential medium (α-MEM, Gibco, United States), supplemented with 10% fetal bovine serum (FBS, Gibco, United States) and 1% penicillin-streptomycin (P/S, Gibco, United States), in a 5% CO_2_ incubator at 37°C. The culture medium was refreshed bi-daily.

All scaffolds were sterilized using a cobalt-60 source with a dosage of 15 kGy. Each scaffold group was weighed and then immersed in complete medium (CM, containing 10% FBS and 1% penicillin-streptomycin) at a concentration of 0.1 g/mL for 24 h. The extractions were subsequently filtered through 0.22 μm filters and stored at 4°C until further use. Cell proliferation of the scaffolds was assessed using the CCK-8 assay. MC3T3-E1 cells were seeded in a 96-well plate at a density of 3 × 10^3^ cells per well. After 24 h, the original medium was replaced. For the control group, α-MEM complete medium without the extractions was used, while the experimental group received the extraction-medium, with medium changes occurring every 3 days. Culture medium was aspirated on days 1, 3, and five of cultivation, and cells were treated with 10 μL of CCK-8 and 100 μL of fresh complete medium. After incubating for 1 h, optical density (OD) values were measured at a wavelength of 450 nm using a Enzyme Marker (Biorad × Mark™, United States). Cell proliferation was determined by comparing OD values.

### 2.10 Cell viability assay

Cytotoxicity was evaluated using a live/dead cell staining kit (Beyotime, China). Initially, MC3T3-E1 cells were seeded in a 96-well plate at a density of 3 × 10^3^ cells per well, with three replicates per condition (*n* = 3). After 24 h, the medium in the control group was refreshed with complete medium, while the experimental groups received various concentrations of scaffold extractions. On days 1 and 5, the medium and extracts were replaced with 100 μL of live/dead cell staining reagent and incubated at room temperature for 30 min. The stained cells were then observed and imaged using a TI2 inverted fluorescence microscope (Nikon, Japan). These images facilitated the analysis and quantification of cell viability and survival rates, enabling assessment of the different treatment groups’ impacts on cell growth.

### 2.11 Cell osteogenic differentiation study

For the osteogenic differentiation assay, MC3T3-E1 cells were cultured in two types of media: pure osteogenic induction medium (ODM) and osteogenic induction media prepared with extractions from each scaffold group. Both media formulations included 50 ug/mL ascorbic acid, 10 mM dexamethasone, and 10 mM β-glycerophosphate (Solarbio, China). The media were refreshed every 3 days. The group cultured in pure ODM served as the control, while those cultured in scaffold-derived osteogenic induction media constituted the experimental groups.

#### 2.11.1 ALP activity assay

Alkaline phosphatase (ALP) activity in MC3T3-E1 cells was assessed using a BCIP/NBT staining kit. Cells were seeded at a density of 1 × 10^5^ cells per well in a 6-well plate and, once adhered, were cultured in osteogenic induction medium containing extractions. On Days 7 and 14, the medium was removed, and the cells were washed with PBS, fixed with 4% paraformaldehyde at room temperature, and washed again. The staining solution, prepared as per the kit instructions, was added (400 μL per well) and incubated in the dark at room temperature for 30 min. The reaction was halted by washing with PBS, and the stained cells were observed and imaged using a TI2 inverted fluorescence microscope (Nikon, Japan). Cells cultured in osteogenic induction medium served as the control group.

#### 2.11.2 Alizarin red staining

Experiments were conducted following the protocol for Alizarin Red Staining solution (Beyotime, China). Cells underwent a culture period of 14 and 21 days as outlined in Section 2.11.1. They were then fixed with 4% paraformaldehyde for 10 min and washed three times with PBS. An adequate volume of Alizarin Red Staining solution was subsequently applied, and the cells were incubated for 30 min. Staining was terminated using distilled water. Finally, calcium deposits were examined with a Nikon TI2 inverted fluorescence microscope (Nikon, Japan).

#### 2.11.3 Western blotting

The cells were seeded and cultured on scaffolds for durations of 7 and 14 days. Subsequently, the cells were harvested to analyze the synthesis of OSX, OCN, OPN, and Runx2 proteins using Western blotting.

### 2.12 Statistical analysis

Data analyses were conducted using SPSS 26.0, while graphical representations were created with GraphPad Prism 8 and Origin 2021. Data are presented as mean ± standard deviation. Overall comparisons utilized one-way analysis of variance (ANOVA), and inter-group differences were assessed using the least significant difference (LSD) method. Statistical significance was established at *p < 0.05*. Levels of significance for *p < 0.05* and *p < 0.01* are indicated by “*” and “**”.

## 3 Result

### 3.1 FTIR analysis

**FIGURE 1:**
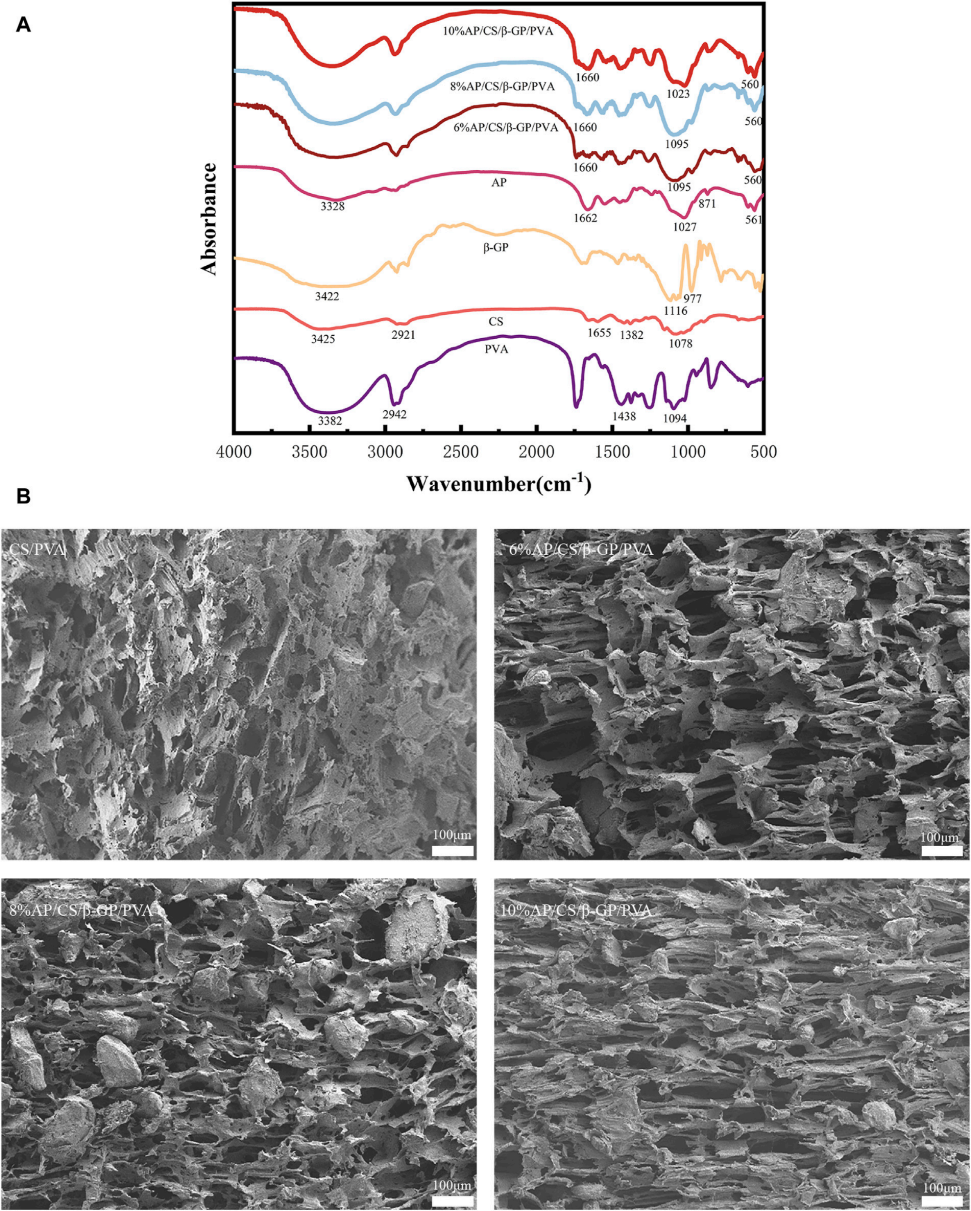
**(A)** The FTIR spectra of hydrogel scaffold and its components. **(B)** Microscopic structure of each group of scaffold under scanning electron microscope: CS/PVA, 6%AP/CS/β-GP/PVA, 8%AP/CS/β-GP/PVA, 10%AP/CS/β-GP/PVA.

To investigate the composition and structural alterations of the hydrogel scaffold, Fourier Transform Infrared Spectroscopy (FTIR) was employed to examine the individual and combined effects of AP, CS, β-GP, PVA, and the AP/CS/β-GP/PVA composite. Figure 1A illustrates that FTIR analysis identified a minor peak at 3,328 cm^−1^ in AP, indicative of O–H vibrations from water molecules, with phosphate peaks at 1,027 cm^−1^ and 561 cm^−1^. Notably, carbonated hydroxyapatite displayed characteristic peaks at 1,662 cm^−1^ and 871 cm^−1^. The PVA spectrum featured a broad band from 3,700 to 3,180 cm^−1^ due to O–H bond stretching, and a peak at 2,941 cm^−1^ associated with C–H bond stretching. Additionally, peaks at 1,438 cm^−1^ and 1,094 cm^−1^ corresponded to CH2 group wagging and C–OH bond stretching, respectively. The chitosan spectrum revealed a broad band between 3,500 cm^−1^ and 3,000 cm^−1^ due to overlapping OH and NH group stretches, with a distinct peak at 2,921 cm^−1^ for asymmetric C-H bond stretching and another at 1,078.68 cm^−1^ for C-O-C group vibrations. Amide peaks at 1,382 cm^−1^ and 1,655 cm^−1^ represented N–H stretching and C=O stretching, respectively. β-GP displayed characteristic peaks at 3,422 cm^−1^ for the −OH bond, 977 cm^−1^ for the P-OH bond, and 1,116 cm^−1^ for the P=O bond. The FTIR spectrum of the hydrogel scaffold highlighted peaks characteristic of all components, suggesting primary interaction through hydrogen bonding without substantial chemical reactions, although some peak shifts occurred. Notably, the peak at 3,425 cm^−1^ intensified and shifted to a lower wavenumber, indicating enhanced intermolecular hydrogen bonding within the composite, contributing to a denser structure with higher cohesion strength. Moreover, the hydrogel scaffold showed distinct peaks of AP and PVA at 1,095, 1,023, 1,660, and 560 cm^−1^, affirming the effective synthesis of the scaffold through a straightforward and reliable method.

### 3.2 Microstructure of scaffolds

The structural characteristics and applications of scaffolds are intimately linked to their architecture. The organization and density of the structure influence the scaffolds’ strength and the complexity of dehydration processes. The number and dimensions of pores impact the swelling capacity, equilibrium moisture content, and the efficacy of nutrient and cellular product transport (Kołodziejska et al., 2023). Scanning electron microscopy observations (Figure 1B) reveal that hydrogel scaffolds exhibit high porosity and an interconnected pore network formed through solvent freezing and freeze-drying techniques, featuring uniformly distributed pores. Following the incorporation of AP, there is a significant enlargement of pores relative to the control group, indicating that AP particles are uniformly dispersed throughout the pores. Additionally, increasing AP concentrations modify the pore morphology, particularly in scaffolds with 10% AP, suggesting that excessive AP can disrupt the interaction between chitosan and β-glycero phosphate.

### 3.3 Wettability analysis

**FIGURE 2:**
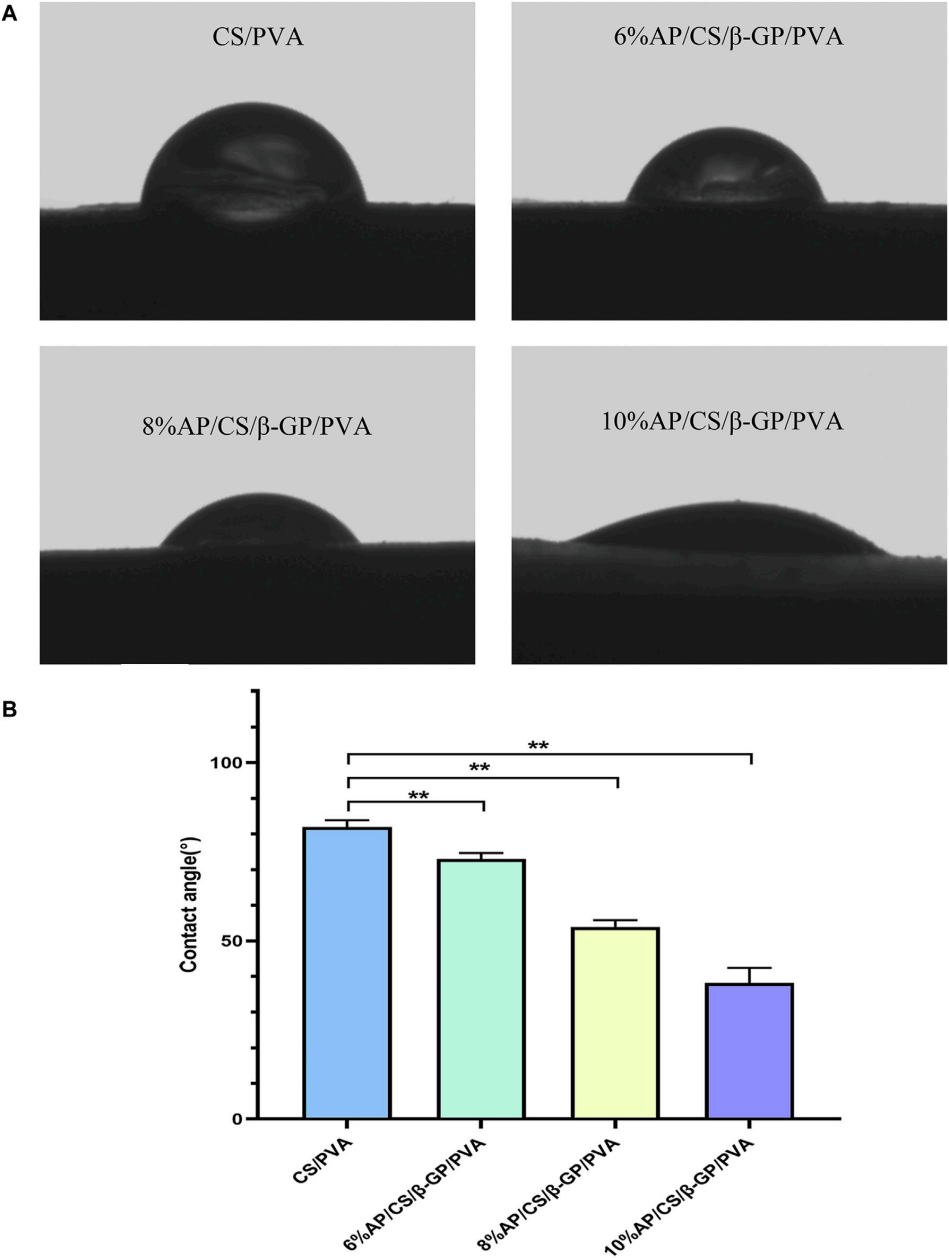
**(A)** Hydrophilicity of scaffolds was detected by water contact angle meter. **(B)** Comparison of contact angles of hydrogel scaffolds in various groups (*p < 0.05, **p < 0.01).

Contact angle measurements are frequently utilized to evaluate the hydrophilicity of materials, which is crucial for promoting cell adhesion and migration. In our study, we performed these measurements on various scaffolds to determine the influence of AP content, as shown in Figure 2A. The contact angles for the CS/PVA, 6% AP/CS/β-GP/PVA, 8% AP/CS/β-GP/PVA, and 10% AP/CS/β-GP/PVA hydrogel scaffolds were recorded at 81.89° ± 1.57°, 72.99° ± 1.32°, 53.90° ± 1.54°, and 38.27° ± 3.40°, respectively. Notably, a decrease in contact angle was observed with increasing AP content, indicating enhanced hydrophilicity (Figure 2B). This increase in hydrophilicity, likely attributed to the augmented surface roughness from higher AP concentrations, enhances cell adhesion and proliferation.

### 3.4 Porosity of scaffolds

**FIGURE 3:**
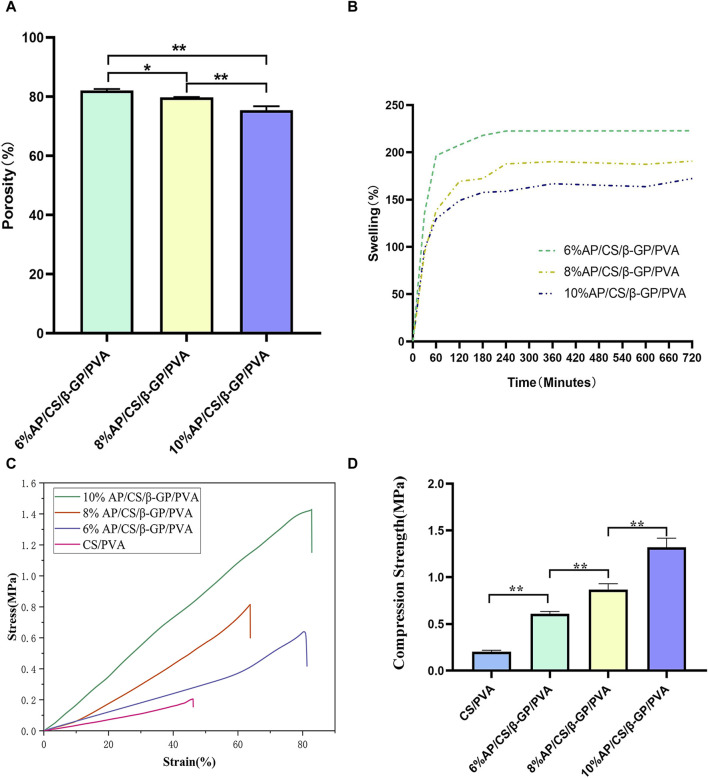
**(A)** Comparison of porosity rates of hydrogel scaffolds in each group. **(B)** Comparison of Swelling rates of hydrogel scaffolds in each group. **(C)** Stress-strain curves of hydrogel scaffolds in each group. **(D)** Compression strength of hydrogel scaffolds in each group (*p < 0.05, **p < 0.01).

As depicted in Figure 3A, our analysis of hydrogel scaffolds with varying AP contents revealed that the porosities of 6%AP/CS/β-GP/PVA, 8%AP/CS/β-GP/PVA, and 10%AP/CS/β-GP/PVA hydrogel scaffolds are 82.1% ± 0.4%, 79.7% ± 0.12%, and 75.4% ± 1.3%, respectively. Notably, an inverse correlation exists between AP content and porosity, showing a statistically significant decreasing trend (*p < 0.05*). This reduction in porosity might be attributed to the excessive AP filling the voids within the hydrogel scaffold. Furthermore, high-porosity scaffolds are advantageous for enhancing healing outcomes, as they facilitate cell migration and angiogenesis, thereby creating optimal conditions for new bone formation.

### 3.5 Swelling property of scaffolds

This study investigates the impact of varying concentrations of AP on the swelling properties of scaffolds. Observational data from Figure 3B shows that the swelling rate of all scaffolds increases rapidly within the first 60 min. Subsequently, from 60 to 240 min, the swelling rate significantly decelerates and stabilizes from 240 to 300 min. Experimental findings indicate that an increase in AP content correlates with a reduction in the swelling rate of the scaffolds. For instance, the swelling percentages of the 6%AP/CS/β-GP/PVA, 8%AP/CS/β-GP/PVA, and 10%AP/CS/β-GP/PVA hydrogel scaffolds are 221.75% ± 2.15%, 185.70% ± 7.65%, and 163.84% ± 6.0%, respectively. Statistically, the differences in swelling rates among these concentrations are not significant (*p > 0.05*).

### 3.6 Mechanical properties of scaffolds

Ideal tissue engineering scaffolds must satisfy the diverse mechanical requirements of tissues. For bone tissue engineering specifically, superior mechanical properties are crucial (Pelin et al., 2023). According to data presented in Figures 3C, D, the maximum compressive strengths for the CS/PVA group, 6%AP/CS/β-GP/PVA group, 8%AP/CS/β-GP/PVA group, and 10%AP/CS/β-GP/PVA group are 0.2037 ± 0.014 MPa, 0.6085 ± 0.025 MPa, 0.8680 ± 0.062 MPa, and 1.3186 ± 0.092 MPa, respectively, showing statistically significant enhancements (*p < 0.05*). These results demonstrate that an increase in antler powder content corresponds to substantial improvements in the mechanical properties of the scaffolds. Furthermore, the formation of numerous hydrogen bonds between PVA and CS also contributes to improved compressive performance.

### 3.7 Cell proliferation analysis

**FIGURE 4:**
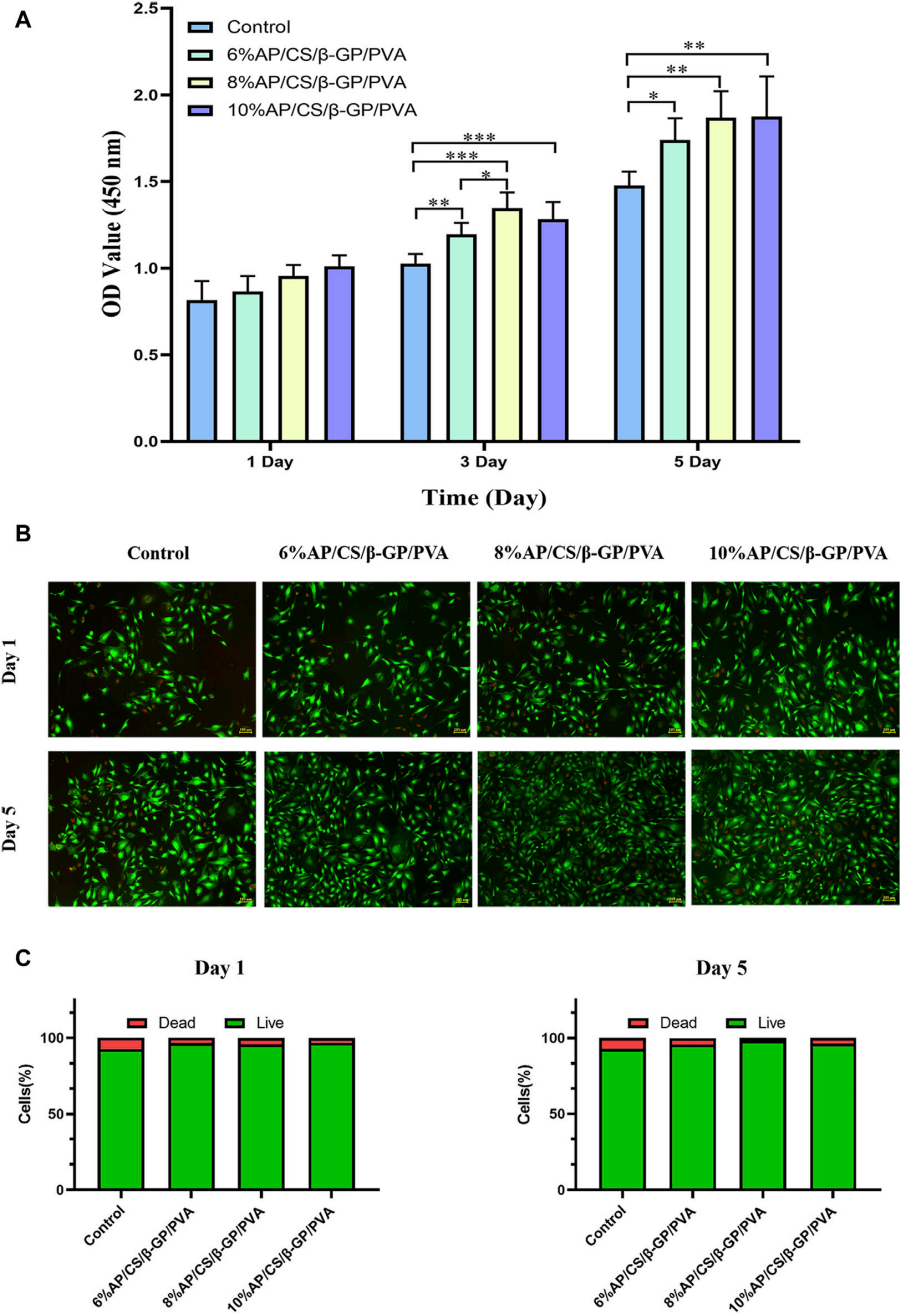
**(A)** The effect of hydrogel scaffolds in each group on the proliferation of MC3T3-E1 cells. **(B)** Staining results of live and dead cells of hydrogel scaffolds in each group. **(C)** Proportion of live and dead cells in each group of hydrogel scaffolds (*p < 0.05, **p < 0.01).

In evaluating cell proliferation, the results of the CCK-8 assay are depicted in Figure 4A. Optical density (OD) values, indicative of cell proliferation, exhibit a positive correlation with the increase in cell numbers. Initially, on the first day of the experiment, OD values across all groups were comparable, with no significant differences observed. However, as the culture period extended, the OD values demonstrated a consistent upward trend. By the third and fifth days, the OD values in the experimental groups (6%AP/CS/β-GP/PVA, 8%AP/CS/β-GP/PVA, and 10%AP/CS/β-GP/PVA) were significantly higher than those in the control group (*p < 0.05*), underscoring the beneficial impact of these treatments on cell proliferation. Among these, the groups containing 8% and 10% antler powder displayed the most pronounced effects, whereas the 6% antler powder group showed comparatively weaker effects (*p < 0.05*). Further analysis indicated that there were no significant differences in the promotion of cell proliferation between the 8% and 10% AP groups (*p > 0.05*), suggesting that the optimal enhancement in cell proliferation is likely achieved at approximately 8% antler powder. This observation suggests an optimal concentration threshold for antler powder, above which further increases do not significantly boost cell proliferation. This insight provides critical evidence for determining the effective concentration of antler powder in biomedical applications.

### 3.8 Cell viability analysis

Maintaining a healthy cellular state is essential for comprehensive studies on hydrogel scaffolds and their clinical applications. We further validated the reliability of the CCK-8 assay through a live/dead cell staining test. Figure 4B illustrates the live/dead cell staining results for MC3T3-E1 cells on days 1 and 5, where green fluorescence indicates live cells and red fluorescence indicates dead cells. Compared to the control group, the experimental group exhibited enhanced survival rates and growth dynamics, although the ratio of live to dead cells remained largely stable (refer to Figure 4C). This supports the hydrogel scaffolds’ potential to promote cell proliferation, aligning with prior experimental results on cell proliferation.

### 3.9 Alkaline phosphatase activity

**FIGURE 5:**
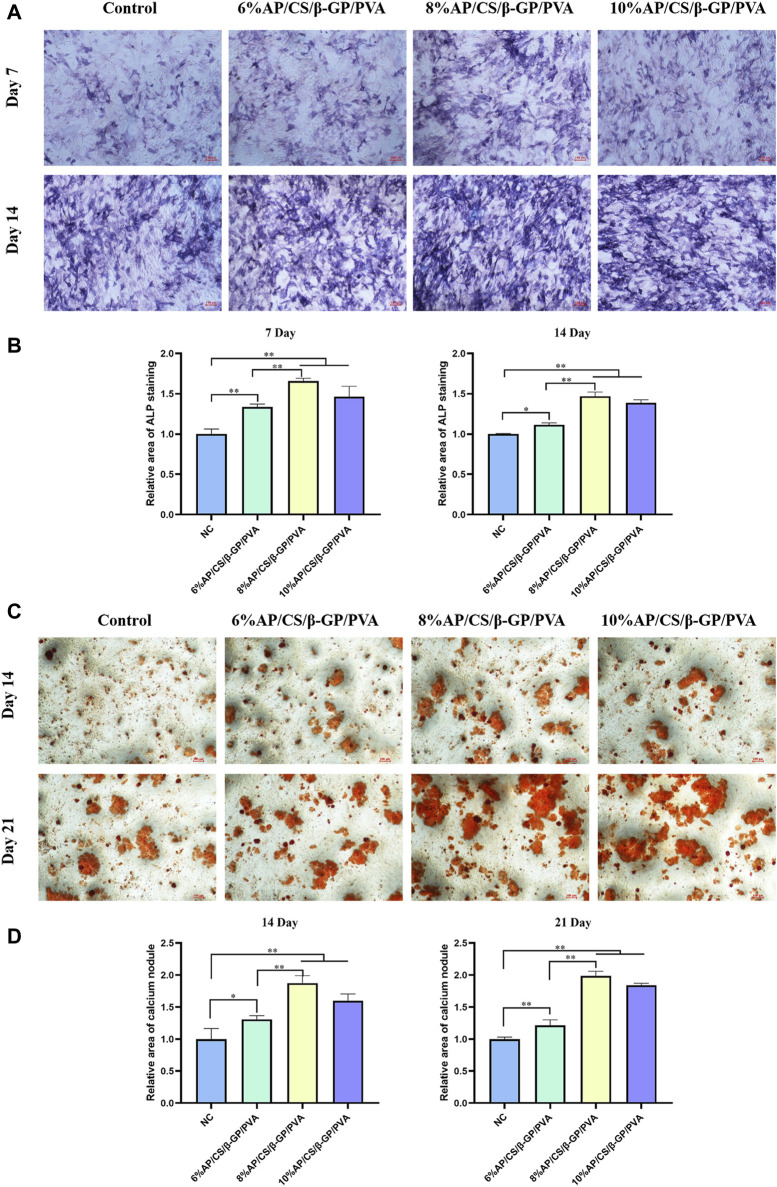
**(A)** ALP staining plots of hydrogel scaffolds for each group. **(B)** Quantitative analysis of ALP staining of hydrogel scaffolds for each group at 7 days, 14 days. **(C)** ARS staining plots of hydrogel scaffolds for each group. **(D)** Quantitative analysis of ARS staining of hydrogel scaffolds for each group at 14 days, 21 days (*p < 0.05, **p < 0.01).

Alkaline phosphatase (ALP) activity serves as a crucial marker of early osteoblastic differentiation, peaking during this phase (Zhang et al., 2023b). It notably increases the accumulation of phosphate and calcium ions in nucleation areas as calcification begins. ALP facilitates this process by enhancing the local concentration of phosphate ions through the hydrolysis of phosphoesters, thus promoting the mineralization of the extracellular matrix (Zhou et al., 2023). As depicted in Figures 5A, B, ALP activity progressively increased in all groups over time. Notably, the antler powder hydrogel group exhibited a distinct purple reaction, indicative of heightened ALP activity. Specifically, on days 7 and 14, the color reaction was more pronounced in the 8%AP/CS/β-GP/PVA and 10%AP/CS/β-GP/PVA groups compared to earlier groups, suggesting that the antler powder scaffold markedly enhances the early osteogenic differentiation of MC3T3-E1 cells.

### 3.10 Alizarin red staining analysis

To further examine the impact of antler powder hydrogel scaffolds on mineralization, this study also monitored the formation of calcium nodules. Figures 5C, D illustrates the calcium nodules identified on days 14 and 21 using the Alizarin Red S (ARS) staining method. The findings indicate that, in comparison to the control group, the experimental group demonstrated a significant increase in both the number and size of calcium nodules. Notably, by day 21, samples containing 8%AP/CS/β-GP/PVA and 10%AP/CS/β-GP/PVA not only exhibited a greater number of nodules but also displayed prominent clusters of calcium nodules.

### 3.11 Osteogenesis-related protein expression analysis

To thoroughly validate the efficacy of hydrogel scaffolds in promoting the differentiation of MC3T3-E1 cells into osteoblasts, this study conducted a detailed evaluation of the expression levels of key osteogenesis-related proteins including RUNX2, OCN, OSX, and OPN. These proteins are vital not only for osteoblast maturation but also for the mineralization process (An et al., 2016). Elevated levels of RUNX2, OCN, OSX, and OPN not only demonstrate the effectiveness of osteogenic differentiation but also suggest the potential of hydrogel scaffolds to augment osteogenic functions.

**FIGURE 6:**
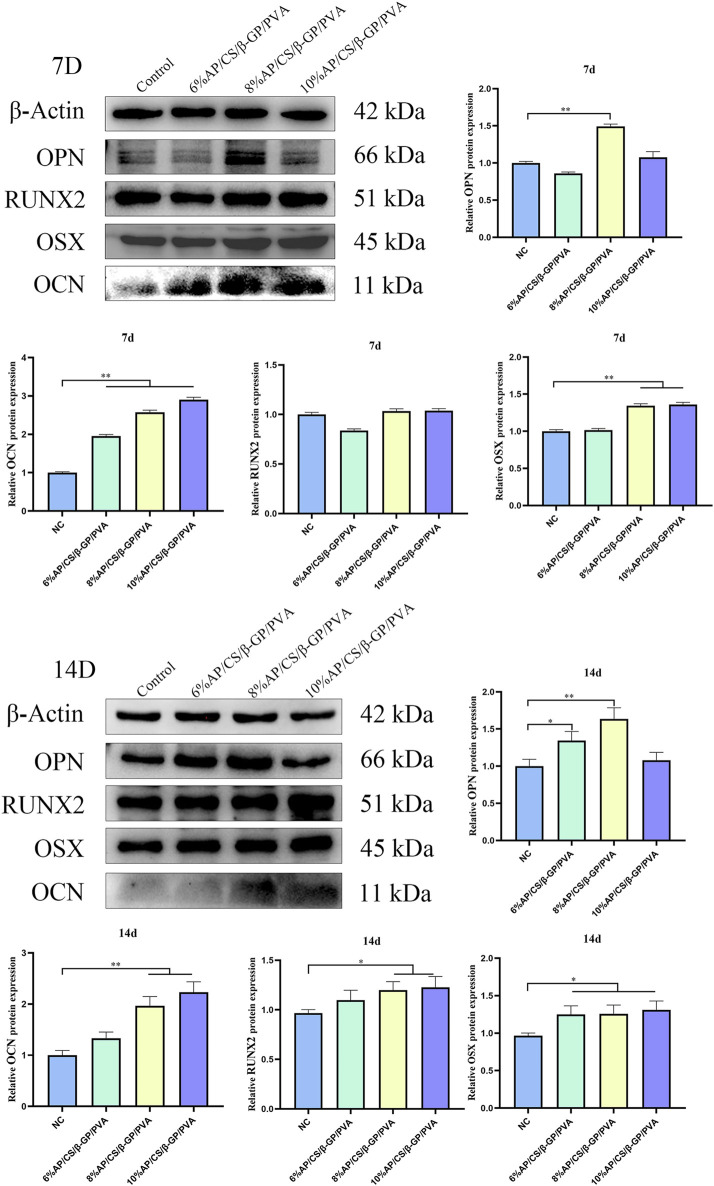
Western blotting strips and relative expression of OCN, OPN, RUNX2 and OSX at 7 days and 14 days (*p < 0.05, **p < 0.01).

The expression levels of RUNX2, OCN, OSX, and OPN are detailed in Figure 6. On day 7, scaffolds containing 8% antler powder exhibited significantly higher OPN levels compared to the control group (*p < 0.05*), and those with 8% and 10% antler powder showed increased OSX expression (*p < 0.05*). Additionally, scaffolds with 6%, 8%, and 10% antler powder demonstrated elevated OCN levels (*p < 0.05*). By day 14, scaffolds with 6% and 8% antler powder displayed higher OPN expression (*p < 0.05*), while those with 6%, 8%, and 10% antler powder had increased OSX levels (*p < 0.05*). Moreover, scaffolds with 8% and 10% antler powder showed significantly higher expressions of both OCN and RUNX2 (*p < 0.05*). Compared to the control, the hydrogel scaffolds containing 8% and 10% antler powder had substantially higher synthesis of RUNX2, OCN, OSX, and OPN proteins. Therefore, the differentiation of MC3T3-E1 cells in these scaffolds may be facilitated by the upregulated expression of RUNX2, OCN, OSX, and OPN genes.

## 4 Discussion

Deer antlers are rich in inorganic elements, notably calcium (Ca), phosphorus (P), and magnesium (Mg). Calcium and phosphorus are crucial for cellular functions, nerve transmission, muscle contraction, blood coagulation, and are intimately linked to human bone metabolism (Ren et al., 2019). Magnesium significantly enhances bone and blood vessel formation (Hohenbild et al., 2021). In Zhang’s research (Zhang et al., 2012), calcined deer antler demonstrated a highly porous structure, with pore sizes ranging from 300 to 600 μm, analogous to human cancellous bone, which has pore sizes of 300 to 800 μm. High-resolution electron microscopy revealed that its structure closely resembles hydroxyapatite crystals, indicating its potential as a superior bone substitute material. Consequently, this study successfully developed a chitosan hydrogel scaffold incorporating deer antler powder, and validated its material characteristics and biological performance. This research aims to create bone tissue engineering materials that exhibit both excellent mechanical properties and biocompatibility.

From a microscopic perspective, electron microscopy and porosity studies of the AP/CS/β-GP/PVA hydrogel scaffolds revealed that the addition of antler powder and β-GP transformed the structure from irregular to a three-dimensional porous configuration with interconnected, loose pores. This change likely results from the formation of hydrogen bonds between CS and β-GP. Notably, increasing the AP content influences pore morphology, particularly in scaffolds with 10% AP content, indicating that excessive AP may disrupt the interaction between chitosan and β-GP. Moreover, the porosities of hydrogel scaffolds with 6%, 8%, and 10% AP content are 82.1% ± 0.4%, 79.7% ± 0.12%, and 75.4% ± 1.3%, respectively, demonstrating a decreasing trend in porosity with increasing AP content (*p < 0.05*). Optimal porosity for tissue engineering scaffolds ranges between 60% and 90%, with pore sizes from 100 to 700 μm (Trifonov et al., 2024). These structures support vascular formation and bone ingrowth, and facilitate nutrient diffusion and transport, thus enhancing cell adhesion, proliferation, and differentiation. Conversely, excessively small pores can impede essential nutrient and oxygen transfer, potentially hindering the integration of bone implants with surrounding bone tissue (Lutzweiler et al., 2020).

FTIR serves as an ideal tool for analyzing the components of tissue engineering materials (Samie et al., 2023). In this study, comparisons of the spectra from AP/CS/β-GP/PVA hydrogel scaffolds with those of pure materials reveal that the composites retain characteristic peaks of all constituents. This suggests that the materials predominantly bond through hydrogen interactions without significant chemical reactions, despite some observed peak shifts. Notably, the peak at 3,425 cm^−1^ both intensified and shifted to a lower wavenumber, suggesting enhanced intermolecular hydrogen bonding within the composite scaffolds. This bonding likely contributes to a denser structure with increased cohesive strength. Additionally, the scaffolds exhibited distinctive vibrational peaks of AP and PVA at 1,095 cm^−1^, 1,023 cm^−1^, 1,660 cm^−1^, and 560 cm^−1^, confirming the successful synthesis of the composite using a straightforward and reliable method.

Hydrophilicity is a critical parameter for assessing the surface performance of biomaterials, as it directly influences their interaction with biological systems, particularly in terms of cell adhesion, proliferation, and migration (Anjum et al., 2023). Hydrophilic surfaces are advantageous because they can more effectively mimic the natural cellular growth environment, thereby providing an optimal interface for cell growth (Wang et al., 2008). The hydrophilicity of scaffold materials is typically evaluated using the contact angle measurement, which is the angle a liquid droplet forms on a solid surface. This method offers an intuitive way to determine the material’s surface characteristics—materials with a contact angle greater than 90° are deemed hydrophobic, indicating that liquids do not readily spread across their surfaces. Conversely, materials with a contact angle less than 90° demonstrate good hydrophilicity, meaning they are easily wetted by liquids (Li et al., 2013). In this experiment, the contact angles on the hydrogel scaffolds containing 6%, 8%, and 10% AP/CS/β-GP/PVA showed a decreasing trend with increasing AP content, suggesting an enhancement in hydrophilicity, which supports improved cell adhesion and proliferation.

The compressive mechanical properties of the scaffolds were evaluated, and a significant increase in compressive strength was noted following the addition of AP. Specifically, the compressive strengths of the hydrogel scaffolds containing 6%, 8%, 10%, and higher percentages of AP/CS/β-GP/PVA were recorded at 0.2037 ± 0.014 MPa, 0.6085 ± 0.025 MPa, 0.8680 ± 0.062 MPa, and 1.3186 ± 0.092 MPa, respectively, all showing statistically significant differences. Additionally, in the AP/CS/β-GP/PVA hydrogel scaffolds, the formation of hydrogen bonds between chitosan and polyvinyl alcohol further enhanced the physical stability and mechanical properties of the hydrogel scaffolds, ensuring reliable performance for biomedical applications.

The biocompatibility of scaffold materials is essential for their successful clinical application, particularly in facilitating cell adhesion, migration, and proliferation, which are foundational for tissue engineering. The CCK-8 proliferation assay demonstrated that the OD values for the AP/CS/β-GP/PVA hydrogel scaffold group were significantly higher than those of the control group, suggesting a favorable effect on cell proliferation. Further analysis indicated no significant difference in promoting cell proliferation between the 8% and 10% AP content, implying that the optimal effect is achieved with approximately 8% antler powder. Additionally, live/dead cell staining results, consistent with the CCK-8 findings, showed that the ratio of live to dead cells remained stable on the first and fifth days of co-culturing with the scaffolds, confirming the absence of significant toxicity to MC3T3-E1 cells. In Zhang’s study (Zhang et al., 2013), various bioactive factors, including icariin, deer antler peptides, and recombinant human bone morphogenetic protein-2, were loaded onto calcined deer antler cancellous bone composites and co-cultured with rat bone marrow mesenchymal stem cells. The outcomes revealed no noticeable toxicity and an ability to promote proliferation, aligning with the findings of this study.

In this experiment, in addition to assessing cytotoxicity and proliferative capacity, we focused on evaluating the osteogenic differentiation of MC3T3-E1 cells induced by the composite scaffold. ALP is an enzyme secreted during the differentiation of osteoprogenitor cells and is considered an early biomarker of bone formation (Li et al., 2024). The level of ALP activity directly mirrors the osteogenic potential of the cells (Liu et al., 2024). In this study, The scaffold group containing deer antler powder exhibited a pronounced purple reaction, indicative of enhanced ALP activity. Notably, on days 7 and 14, the color intensity in the 8% and 10% AP/CS/β-GP/PVA groups was significantly stronger than in earlier measurements. These findings suggest that the deer antler powder hydrogel scaffold markedly enhances early osteogenic differentiation in MC3T3-E1 cells. Simultaneously, mineralized nodules act as indicators of osteoblast differentiation and maturation (Krause et al., 2011). In our study, these nodules were stained with ARS, revealing that the deer antler powder scaffold group exhibited significantly larger areas of calcium nodule aggregation than the control group at both 14 and 21 days, demonstrating superior osteogenic capability. Furthermore, in Cong’s study (Ren et al., 2019), deer antler water extract (PAAE) was isolated and purified to explore its effects on bone marrow mesenchymal stem cells (BMSCs). The results indicated that PAAE could enhance the proliferation and osteoblastic differentiation of BMSCs, accompanied by increased ALP activity and extracellular matrix mineralization. These findings are consistent with those of our experiment.

To further validate the osteogenic ability of AP/CS/β-GP/PVA hydrogel scaffold materials, we analysed the expression of osteogenesis-related genes in MC3T3-E1 cells from different experimental groups by Western blot. Runx2, a crucial transcription factor involved in osteogenic differentiation and bone formation, promotes gene expression during the early stages of osteogenesis (Li et al., 2023). Osx is a novel zinc-finger-containing osteoblast-specific transcription factor that is critical for the proliferation, differentiation, and formation of osteoblasts (Fu et al., 2018). During bone formation, OPN is produced by osteoblasts and osteoclasts and is one of the more abundant non-collagen proteins in the bone matrix (Wu et al., 2019). OCN appears in the late stages of osteoblast differentiation. Ducy et al. (1996) noted that OCN can bind with Ca^2+^ to regulate calcium homeostasis and bone mineralization. These factors collectively drive the development of bone cells and the formation of bone tissue. In our experiments, on days 7 and 14 of osteogenic induction, the expression levels of RUNX2, OCN, OSX, and OPN in the AP/CS/β-GP/PVA hydrogel scaffold group were superior to those in the control group, confirming that the AP/CS/β-GP/PVA hydrogel scaffold enhances mineralization and differentiation in MC3T3-E1 cells.

Based on our findings, the AP/CS/β-GP/PVA hydrogel scaffolds demonstrate varying degrees of efficacy depending on the concentration of AP (Table 1). The results indicate that scaffolds with higher AP content (8% and 10%) exhibit improved mechanical properties, hydrophilicity, and biocompatibility, which are crucial for bone tissue engineering applications. The 8% AP/CS/β-GP/PVA hydrogel scaffold formulation appears to offer the best balance between mechanical strength and biocompatibility. This formulation provides optimal hydrophilicity, mechanical strength, and supports cell proliferation and differentiation effectively. While the 10% AP/CS/β-GP/PVA formulation offers superior mechanical strength, its reduced porosity and swelling capacity may limit nutrient and oxygen transfer, potentially affecting cell viability and integration.

**TABLE 1:** Summary of results for each formulation.

Scaffold formulation	Porosity (%)	Hydrophilicity (contact Angle°)	Swelling rate (%)	Compressive strength (MPa)	Cell proliferation (OD 450 nm)	ALP activity	Calcium nodule formation	Osteogenesis-related protein expression	Advantages
6%AP/CS/β-GP/PVA	82.1 ± 0.4	72.99 ± 1.32	185.70 ± 7.65	0.6085 ± 0.025	Increased	Moderate	Moderate	Increased	Enhanced mechanical properties, Moderate cell proliferation and differentiation
8%AP/CS/β-GP/PVA	79.7 ± 0.12	53.90 ± 1.54	163.84 ± 6.0	0.8680 ± 0.062	Optimal	High	High	Significant increase	Optimal cell proliferation and differentiation, Balanced properties
10%AP/CS/β-GP/PVA	75.4 ± 1.3	38.27 ± 3.40	150.0 ± 6.0	1.3186 ± 0.092	High	High	High	Highest mechanical strength	Maximum mechanical strength, Highest osteogenic potential

This study successfully synthesized a three-dimensional hydrogel scaffold with a loose, porous structure using deer antler powder, chitosan, β-glycerophosphate, and polyvinyl alcohol. This scaffold provides an optimal microenvironment for osteoblasts, effectively enhancing their activity. Despite its advantages, the hydrogel scaffold has certain limitations. Cellular behavior was assessed indirectly using conditioned medium rather than direct cell-scaffold interactions. While this approach provided initial insights, it does not fully capture the complex dynamics of cell behavior in direct contact with the scaffold materials. Future studies will include direct contact tests to evaluate how cells adhere to, proliferate on, and differentiate within the AP/CS/β-GP/PVA hydrogel scaffolds. These tests will help elucidate the full potential and biocompatibility of the scaffolds in bone tissue engineering applications. At the same time, the precise mechanisms by which deer antler powder promotes bone formation remain unclear. This discovery paves the way for further *in vivo* experimental studies and clinical applications.

## 5 Conclusion

We have demonstrated the production and materials characterization of porous AP/CS/β-GP/PVA hydrogel scaffolds. The study’s results indicated that an increase in the content of deer antler powder significantly enhanced both the hydrophilicity and physical properties of the hydrogel scaffold, although there was a slight reduction in porosity. Cell culture experiments further verified that scaffolds with higher deer antler powder content were more conducive to the proliferation and differentiation of MC3T3-E1 cells. This effect likely results from the enhanced expression of genes such as RUNX2, OCN, OSX, and OPN, which are essential for cellular differentiation. Consequently, the AP/CS/β-GP/PVA hydrogel scaffolds demonstrate potential as highly promising biomaterials for bone tissue engineering.

## Data Availability

The raw data supporting the conclusion of this article will be made available by the author, without undue reservation.

## References

[B1] Ahmadi R. de Bruijn J. D. (2008). Biocompatibility and gelation of chitosan-glycerol phosphate hydrogels. J. Biomed. Mater. Res. Part A 86 (3), 824–832. 10.1002/jbm.a.31676 18041728

[B2] An J. Yang H. Zhang Q. Liu C. Zhao J. Zhang L. (2016). Natural products for treatment of osteoporosis: the effects and mechanisms on promoting osteoblast-mediated bone formation. Life Sci. 147 (February), 46–58. 10.1016/j.lfs.2016.01.024 26796578

[B3] Anjum S. Kumar Arya D. Saeed M. Ali D. Athar M. S. Yulin W. (2023). Multifunctional electrospun nanofibrous scaffold enriched with alendronate and hydroxyapatite for balancing osteogenic and osteoclast activity to promote bone regeneration. Front. Bioeng. Biotechnol. 11 (November), 1302594. 10.3389/fbioe.2023.1302594 38026845 PMC10665524

[B4] Arpornmaeklong P. Jaiman N. Apinyauppatham K. Fuongfuchat A. Boonyuen S. (2023). Effects of calcium carbonate microcapsules and nanohydroxyapatite on properties of thermosensitive chitosan/collagen hydrogels. Polymers 15 (2), 416. 10.3390/polym15020416 36679297 PMC9861171

[B5] Badr A. M. Shalaby H. K. Awad M. A. Hashem M. A. (2023). Assessment of bone morphogenetic protein-7 loaded chitosan/β-Glycerophosphate hydrogel on periodontium tissues regeneration of class III furcation defects. Saudi Dent. J. 35 (6), 760–767. 10.1016/j.sdentj.2023.05.027 37817788 PMC10562118

[B6] Bernardi S. Macchiarelli G. Bianchi S. (2020). Autologous materials in regenerative dentistry: harvested bone, platelet concentrates and dentin derivates. Mol. (Basel, Switzerland) 25 (22), 5330. 10.3390/molecules25225330 PMC7696510 33203172

[B7] Bouziane A. Hamdoun R. Abouqal R. Ennibi O. (2020). Global prevalence of aggressive periodontitis: a systematic review and meta-analysis. J. Clin. Periodontology 47 (4), 406–428. 10.1111/jcpe.13266 32011029

[B8] Chu W. Hu G. Lin P. Zhang W. Ma Z. (2021). The use of a novel deer antler decellularized cartilage-derived matrix scaffold for repair of osteochondral defects. J. Biol. Eng. 15 (1), 23. 10.1186/s13036-021-00274-5 34479610 PMC8414868

[B9] Cutiongco M. F. A. Goh S. H. Aid-Launais R. Le Visage C. Low H. Y. Yim E. K. F. (2016). Planar and tubular patterning of micro and nano-topographies on poly(vinyl alcohol) hydrogel for improved endothelial cell responses. Biomaterials 84 (April), 184–195. 10.1016/j.biomaterials.2016.01.036 26828683

[B10] Ducy P. Desbois C. Boyce B. Pinero G. Story B. Dunstan C. (1996). Increased bone formation in osteocalcin-deficient mice. Nature 382 (6590), 448–452. 10.1038/382448a0 8684484

[B11] Fu X. Li Y. Huang T. Yu Z. Ma K. Yang M. (2018). Runx2/Osterix and zinc uptake synergize to orchestrate osteogenic differentiation and citrate containing bone apatite formation. Adv. Sci. (Weinheim, Baden-Wurttemberg, Germany) 5 (4), 1700755. 10.1002/advs.201700755 PMC5908346 29721422

[B12] Gillman C. E. Jayasuriya A. C. (2021). FDA-approved bone grafts and bone graft substitute devices in bone regeneration. Mater. Sci. Eng. C, Mater. Biol. Appl. 130 (November), 112466. 10.1016/j.msec.2021.112466 34702541 PMC8555702

[B13] Hohenbild F. Arango Ospina M. Schmitz S. I. Moghaddam A. Boccaccini A. R. Westhauser F. (2021). An *in vitro* evaluation of the biological and osteogenic properties of magnesium-doped bioactive glasses for application in bone tissue engineering. Int. J. Mol. Sci. 22 (23), 12703. 10.3390/ijms222312703 34884519 PMC8657676

[B14] Huang X. Lou Y. Duan Y. Liu He Tian J. Shen Ya (2024). Biomaterial scaffolds in maxillofacial bone tissue engineering: a review of recent advances. Bioact. Mater. 33 (March), 129–156. 10.1016/j.bioactmat.2023.10.031 38024227 PMC10665588

[B15] Kloss F. R. Kämmerer P. W. Kloss-Brandstätter A. (2023). First clinical case report of a xenograft–allograft combination for alveolar ridge augmentation using a bovine bone substitute material with hyaluronate (Cerabone® plus) combined with allogeneic bone granules (Maxgraft®). J. Clin. Med. 12 (19), 6214. 10.3390/jcm12196214 37834860 PMC10573600

[B16] Kołodziejska B. Figat R. Kolmas J. (2023). Biomimetic apatite/natural polymer composite granules as multifunctional dental tissue regenerative material. Int. J. Mol. Sci. 24 (23), 16751. 10.3390/ijms242316751 38069072 PMC10706555

[B17] Krause U. Seckinger A. Gregory C. A. (2011). Assays of osteogenic differentiation by cultured human mesenchymal stem cells. Methods Mol. Biol. (Clifton, N.J.) 698, 215–230. 10.1007/978-1-60761-999-4_17 21431522

[B18] Lan W. Zhang X. Xu M. Zhao L. Huang Di Wei X. (2019). Carbon nanotube reinforced polyvinyl alcohol/biphasic calcium phosphate scaffold for bone tissue engineering. RSC Adv. 9 (67), 38998–39010. 10.1039/c9ra08569f 35540653 PMC9075967

[B19] Li Ai D. Sun Z. Z. Zhou M. Xu X. X. Ma J. Y. Zheng W. (2013). Electrospun chitosan-graft-PLGA nanofibres with significantly enhanced hydrophilicity and improved mechanical property. Colloids Surfaces. B, Biointerfaces 102 (February), 674–681. 10.1016/j.colsurfb.2012.09.035 23107946

[B20] Li C. (2023). Deer antler renewal gives insights into mammalian epimorphic regeneration. Cell. Regen. 12 (July), 26. 10.1186/s13619-023-00169-4 37490254 PMC10368610

[B21] Li C. Suttie J. M. (2000). Histological studies of pedicle skin formation and its transformation to antler velvet in red deer (Cervus elaphus). Anatomical Rec. 260 (1), 62–71. 10.1002/1097-0185(20000901)260:1<62::aid-ar70>3.0.co;2-4 10967537

[B22] Li Q. Wu Y. Zhang Y. Mao J. Zhang Z. (2024). Enhancing osteogenic differentiation of MC3T3-E1 cells during inflammation using UPPE/β-TCP/TTC composites *via* the Wnt/β-catenin pathway. RSC Adv. 14 (3), 1527–1537. 10.1039/d3ra05529a 38179095 PMC10763654

[B23] Li Y. Li J. Jiang S. Cheng Z. Zhao C. Jiao Y. (2023). The design of strut/TPMS-based pore geometries in bioceramic scaffolds guiding osteogenesis and angiogenesis in bone regeneration. Mater. Today Bio 20 (May), 100667. 10.1016/j.mtbio.2023.100667 PMC10238647 37273795

[B24] Liu Y. Shi C. Ming P. Yuan L. Jiang X. Jiang M. (2024). Biomimetic fabrication of Sr-silk fibroin Co-assembly hydroxyapatite based microspheres with angiogenic and osteogenic properties for bone tissue engineering. Mater. Today Bio 25 (February), 101011. 10.1016/j.mtbio.2024.101011 PMC10912917 38445010

[B25] Luo X. Niu J. Su G. Zhou L. Zhang X. Liu Y. (2023). Research progress of biomimetic materials in oral medicine. J. Biol. Eng. 17 (1), 72. 10.1186/s13036-023-00382-4 37996886 PMC10668381

[B26] Lutzweiler G. Halili A. N. Vrana N. E. (2020). The overview of porous, bioactive scaffolds as instructive biomaterials for tissue regeneration and their clinical translation. Pharmaceutics 12 (7), 602. 10.3390/pharmaceutics12070602 32610440 PMC7407612

[B27] Maevskaia E. Guerrero J. Ghayor C. Bhattacharya I. Weber F. E. (2023). Triply periodic minimal surface-based scaffolds for bone tissue engineering: a mechanical, *in vitro* and *in vivo* study. Tissue Eng. 29 (19–20), 507–517. 10.1089/ten.TEA.2023.0033 PMC10611970 37212290

[B28] Pelin I. M. Popescu I. Calin M. Rebleanu D. Voicu G. Ionita D. (2023). Tri-component hydrogel as template for nanocrystalline hydroxyapatite deposition using alternate soaking method for bone tissue engineering applications. Gels (Basel, Switzerland) 9 (11), 905. 10.3390/gels9110905 37998995 PMC10671408

[B29] Ren C. Gong W. Li F. Xie M. (2019). Pilose antler aqueous extract promotes the proliferation and osteogenic differentiation of bone marrow mesenchymal stem cells by stimulating the BMP-2/smad1, 5/Runx2 Signaling pathway. Chin. J. Nat. Med. 17 (10), 756–767. 10.1016/S1875-5364(19)30092-5 31703756

[B30] Samie M. Khan A. F. Rahman S. U. Iqbal H. Yameen M. A. Aqif A. C. (2023). Drug/bioactive eluting chitosan composite foams for osteochondral tissue engineering. Int. J. Biol. Macromol. 229 (February), 561–574. 10.1016/j.ijbiomac.2022.12.293 36587649

[B31] Santos M. S. Dos Santos A. B. Carvalho M. S. (2023). New insights in hydrogels for periodontal regeneration. J. Funct. Biomaterials 14 (11), 545. 10.3390/jfb14110545 PMC10672517 37998114

[B32] Steiner-Bogdaszewska Ż. Tajchman K. Ukalska-Jaruga A. Florek M. Pecio M. (2022). The mineral composition of bone marrow, plasma, bones and the first antlers of farmed fallow deer. Animals Open Access J. MDPI 12 (20), 2764. 10.3390/ani12202764 PMC9597732 36290150

[B33] Sun Q. Zhu Z. Meng F. Zhao R. Xing Li Long X. (2023). Application of a modified osteotomy and positioning integrative template system (MOPITS) based on a truncatable reconstruction model in the precise mandibular reconstruction with fibula free flap: a pilot clinical study. BMC Oral Health 23 (1), 842. 10.1186/s12903-023-03596-6 37940900 PMC10630995

[B34] Tong J. Zhou H. Zhou J. Chen Y. Shi J. Zhang J. (2022). Design and evaluation of chitosan-amino acid thermosensitive hydrogel. Mar. Life Sci. Technol. 4 (1), 74–87. 10.1007/s42995-021-00116-9 37073351 PMC10077161

[B35] Trifonov A. Shehzad A. Mukasheva F. Moazzam M. Akilbekova D. (2024). Reasoning on pore terminology in 3D bioprinting. Gels (Basel, Switzerland) 10 (2), 153. 10.3390/gels10020153 38391483 PMC10887720

[B36] Wang A.-J. Lu Y.-P. Zhu R.-F. Shi-Tong Li Xiao G.-Y. Zhao G.-F. (2008). Effect of sintering on porosity, phase, and surface morphology of spray dried hydroxyapatite microspheres. J. Biomed. Mater. Res. Part A 87 (2), 557–562. 10.1002/jbm.a.31895 18306315

[B37] Wu J. Li N. Fan Y. Wang Y. Gu Y. Li Z. (2019). The conditioned medium of calcined tooth powder promotes the osteogenic and odontogenic differentiation of human dental pulp stem cells via MAPK signaling pathways. Stem Cells Int. 2019 (March), 1–13. 10.1155/2019/4793518 PMC6444228 31015840

[B38] Xu D. Gu C. Dai J. Zhi Li (2021). Bi-layered composite scaffold for repair of the osteochondral defects. Adv. Wound Care 10 (8), 401–414. 10.1089/wound.2019.1140 PMC8236300 33076773

[B39] Xu M. Qin M. Zhang X. Zhang X. Li J. Hu Y. (2020). Porous PVA/SA/HA hydrogels fabricated by dual-crosslinking method for bone tissue engineering. J. Biomater. Sci. Polym. Ed. 31, 816–831. 10.1080/09205063.2020.1720155 31971484

[B40] Zhang C. Zhou Z. Liu N. Chen J. Wu J. Zhang Y. (2023a). Osteogenic differentiation of 3D-printed porous tantalum with nano-topographic modification for repairing craniofacial bone defects. Front. Bioeng. Biotechnol. 11, 1258030. 10.3389/fbioe.2023.1258030 37671184 PMC10475942

[B41] Zhang X. Cai Q. Liu H. Heng B. C. Peng H. Song Y. (2012). Osteoconductive effectiveness of bone graft derived from antler cancellous bone: an experimental study in the rabbit mandible defect model. Int. J. Oral Maxillofac. Surg. 41 (11), 1330–1337. 10.1016/j.ijom.2012.05.014 22704591

[B42] Zhang X. Xu M. Lin S. Yan W. Lin Y. Liu W. (2013). Effects of compatibility of deproteinized antler cancellous bone with various bioactive factors on their osteogenic potential. Biomaterials 34 (36), 9103–9114. 10.1016/j.biomaterials.2013.08.024 24008040

[B43] Zhang Z. He C. Bao C. Li Z. Jin W. Li C. (2023b). MiRNA profiling and its potential roles in rapid growth of velvet antler in gansu red deer (Cervus elaphus Kansuensis). Genes 14 (2), 424. 10.3390/genes14020424 36833351 PMC9957509

[B44] Zhao R. Yang R. Cooper P. R. Khurshid Z. Amin S. Ratnayake J. (2021). Bone grafts and substitutes in dentistry: a review of current trends and developments. Mol. (Basel, Switzerland) 26 (10), 3007. 10.3390/molecules26103007 PMC8158510 34070157

[B45] Zheng D. Tong C. Han L. Lv S. Yin J. Yang K. (2022). Synergetic integrations of bone marrow stem cells and transforming growth factor‐β1 loaded chitosan nanoparticles blended silk fibroin injectable hydrogel to enhance repair and regeneration potential in articular cartilage tissue. Int. Wound J. 19 (5), 1023–1038. 10.1111/iwj.13699 35266304 PMC9284642

[B46] Zheng Y. Lu H. Mu Q. Yi P. Lin L. Pei Li (2023). Effects of sEV derived from SHED and DPSC on the proliferation, migration and osteogenesis of PDLSC. Regen. Ther. 24 (December), 489–498. 10.1016/j.reth.2023.09.009 37767183 PMC10520277

[B47] Zhou J. Zhu Y. Ai D. Zhou M. Han Li Fu Y. (2023). Low-intensity pulsed ultrasound regulates osteoblast-osteoclast crosstalk via EphrinB2/EphB4 signaling for orthodontic alveolar bone remodeling. Front. Bioeng. Biotechnol. 11, 1192720. 10.3389/fbioe.2023.1192720 37425367 PMC10326439

